# Tooth Loss and Risk of Head and Neck Cancer: A Meta-Analysis

**DOI:** 10.1371/journal.pone.0071122

**Published:** 2013-08-19

**Authors:** Ren-Sheng Wang, Xue-Ying Hu, Wan-Jie Gu, Zhen Hu, Bo Wei

**Affiliations:** 1 Department of Radiotherapy, The First Affiliated Hospital, Guangxi Medical University, Nanning, Guangxi, People's Republic of China; 2 School of Public Health, Guangxi Medical University, Nanning, Guangxi, People's Republic of China; 3 Department of Anaesthesiology, The First Affiliated Hospital, Guangxi Medical University, Nanning, Guangxi, People's Republic of China; National Cancer Center, Japan

## Abstract

**Background:**

Observational studies suggest an association between tooth loss and risk of head and neck cancer. However, whether tooth loss is an independent risk factor for head and neck cancer still remains controversial. The aim of this study is to assess the association between tooth loss and head and neck cancer risk.

**Methods:**

Eligible studies were searched in PubMed and Embase databases from their inception to March 2013. A random-effects model or fixed-effects model was used to calculate the overall combined risk estimates.

**Results:**

Eight case-control studies and one cross-sectional study involving 5,204 patients and 5,518 controls were included in the meta-analysis. The overall combined odds ratio for tooth loss and head and neck cancer was 2.00 (95% confidence interval, 1.28–3.14). Similar results yielded both in the moderate and severe tooth loss group. Sensitivity analysis based on various exclusion criteria maintained this significance with respect to head and neck cancer individually. Little evidence of publication bias was observed.

**Conclusion:**

This meta-analysis suggests that tooth loss is associated with increased risk of head and neck cancer. This increase is probably independent of conventional head and neck cancer risk factors.

## Background

Head and neck cancer includes cancer of oral cavity, pharynx, and larynx. It is estimated that one out of 99 people born in the United States today will experience head and neck cancer during their lifetime [1]. It has been recognized as a significant component of the global burden of cancer [2], [3], [4]. Any possible risk factors to increase head and neck cancer should be explored thoroughly. Tobacco use and alcohol consumption have been well established as the predominant etiologic factors for head and neck cancer, with their population-attributable risk for head and neck cancer in the United States by 74% [5]. Other risk factors such as periodontal disease [6], Human papillomavirus (HPV) infections [7], oral hygiene and dental status [8], [9], have also been implicated in the etiology of head and neck cancer.

Periodontal disease is a chronic, destructive disease that affects approximately 35% dentate adults 30 to 90 years old in the United States [10] and up to 90% of the worldwide population [11]. Periodontal disease is believed to be one of the major causes of tooth loss in adults [12]. Nowadays, multiple epidemiologic studies regarding the potential association of tooth loss with head and neck cancer risk have been published [6], [8], [13], [14], [15], [16], [17], [18], [19], [20], [21], [22], [23], [24], [25], [26], [27]. However, these studies have a modest sample size and the evidence has been inconclusive. Therefore, it is highly necessary to conduct a quantitative and systematic summary of the evidence using rigorous methods. At the present study, a meta-analysis was conducted to assess the inconsistent results from published studies on the association of tooth loss with head and neck cancer risk.

## Methods

### Literature search and inclusion criteria

PubMed and Embase databases (from their inception through to March, 2013) were searched to identify relevant studies investigating the association between tooth loss and risk of head and neck cancer. The following search terms were used: *periodontal disease*, *periodontitis*, *tooth loss*, and *head and neck cancer*, filtered by *Human*, without language restrictions. Reference lists of reviews or studies identified in the literature search were hand searched for additional studies. If duplicated data were presented in several studies, only the most recent, largest or most complete study was included. Studies meeting the following criteria were included: (i) evaluating the association between tooth loss and head and neck cancer; (ii) with ≤5 lost teeth as the referent category; and (iii) providing the adjusted odds ratios (ORs) with the corresponding 95%CI or raw data to calculate the crude ORs and 95% confidence intervals (CIs).

### Data extraction and outcome measure

Two reviewers (XYH and WJG) independently extracted data about the characteristics of selected studies using a standardized data extraction form. Data were recorded as follows: first author, year of publication, location, number of subjects (cases/controls), patient characteristics, assessment of tooth loss, cancer site, statistical adjustment for confounding factors, source of controls, study design, and outcome data. Disagreements were resolved by discussion and consensus with a third author (RSW).

### Statistical analyses

OR with 95% CI was used as a common measure of the association between tooth loss and risk of head and neck cancer across studies. For purposes of the present analysis, tooth loss was coded as a three-level indicator variable, with ≤5 lost teeth as the referent category, 6–15 lost teeth as the moderate tooth loss group, and >15 lost teeth as the severe tooth loss group, respectively. Statistical heterogeneity was evaluated using the Cochran *Q* test (significance level at <0.10). The *I* ^2^ statistics [28], which was a quantitative measure of inconsistency across studies, was also calculated. The random-effects model (DerSimonian and Laird method [29]) was taking into account when heterogeneity was observed among studies. Otherwise, a fixed-effects model (Mantel–Haenszel method [30]) was applied. In the presence of heterogeneity, sensitivity analyses based on assessment of tooth loss, adjustment for covariates, and sample size were conducted to identify potential sources. We also assessed the influence of individual studies on the combined risk estimate by sequentially excluding one study in each turn to test the stability of the main results.

Potential publication bias was assessed both by visually inspecting of the Begg funnel plot and statistically via Begg and Egger's unweighted regression tests [31], [32]. A *P* value less than 0.05 was judged as statistically significant, except where otherwise specified. Statistical analyses were carried out with STATA version 11.0 (Stata Corporation, College Station, Texas, USA). All the *P* values were two-sided.

## Results

### Identification of eligible studies

The search strategy identified 3,569 potential studies from PubMed and Embase databases. Of these, the majority were excluded after reviewing titles and abstracts, mainly because they were reviews, letter, comment, or not relevant to our analysis, leaving 18 for full-text review. In the review, 9 articles were excluded for the reasons as follows: three articles with unavailable data for analysis [16], [17], [20], and six articles not with ≤5 lost teeth as the referent category [6], [13], [14], [15], [18], [19]. One study [25] was conducted in Central Europe and Latin America, respectively. We considered it as two case-control studies. Finally, nine studies [8], [21], [22], [23], [24], [25], [26], [27] were included in our meta-analysis. A detailed flowchart of the selection process was shown in **Diagram S1**.

### Study characteristics

The characteristics of all included studies were presented in **Table 1**. Theses studies were published between 2000 and 2010. Sample size ranged from 50 to 1,976 (total 10,722). Eight were case-control studies and one was a cross-sectional prospective study. Among these studies, all reported tooth loss events, eight reported moderate tooth loss events, and eight reported severe tooth loss events.

**Table 1:** Characteristics of studies included in this meta-analysis.

Study	Location	Study design	Group	No. of subjects	Age, Median (Range),yrs	Assessment of tooth loss	Cancer site	Source of controls	Adjustment for covariates
Talamini et al 2000	Italy	Case-control	case	132	60 (27–86)	Self-reported	Oral cavity, oro-pharynx, unspecified cancer		Age, gender, fruit and vegetable intake, and smoking and drinking habits
			control	148	60 (30–83)			Hospital	
Fernandez Garrote et al 2001	Cuba	Case-control	case	200	64 (28–91)	Inspected by dentist	Oral cavity, oro-pharynx, and both site cancer		Age, gender, area of residence, education, smoking and drinking habits
			control	200	62 (25–88)			Hospital	
Balaram et al 2002	India	Case-control	case	591	NA (18–87)	Inspected by interviewer	Oral cavity		Age, center, education and (men only) smoking and drinking habits
			control	582	NA (18–80)			Hospital	
Lissowska et al 2003	Poland	Case-control	case	122	NA (23–80)	Inspected by dentist	Oral cavity, oro-pharynx, unspecified cancer		Age, gender, residence, smoking and drinking habits
			control	124	NA (NA)			Hospital	
Rosenquist et al 2005	Sweden	Case-control	case	132	NA (33–87)	Inspected by dentist	Oral cavity, oro-pharynx, unspecified cancer	Population	NA
			control	320	NA (NA)				
Guha et al 2007	Central Europe	Case-control	case	712	NA (NA)	Inspected by dentist or interviewer	Oral cavity, pharynx, larynx		Age, gender, country, education, tobacco pack-years, cumulative alcohol consumption, and all other oral health variables
			control	928	NA (NA)			Hospital and Population	
Guha et al 2007	Latin America	Case-control	case	1,976	NA (NA)	Inspected by dentist or interviewer	Oral cavity, pharynx, larynx		Age, gender, country, education, tobacco pack-years, cumulative alcohol consumption, and all other oral health variables
			control	1,805	NA (NA)			Hospital and Population	
de Rezende et al 2008	Brazil	cross-sectional	case	50	NA (NA)	Inspected by dentist	Oral cavity, oro-pharynx		NA
			control	50	NA (NA)			Population	
Divaris et al 2010	USA	case-control	case	1,289	NA (20–80)	Self-reported	Oral cavity, pharynx, and larynx		Age, gender, race, education, smoking status and intensity, drinking status, cumulative ethanol consumption, and fruit and vegetable consumption
			control	1,361	NA (20–80)			Population	

NA, not available.

The assessment of tooth loss was from a variety of sources, including self-reported, and inspected by dentist or interviewer. The anatomical sites of head and neck cancer were various across studies, including oral cavity, pharynx, larynx, and unspecified cancer site. Three studies reported an association between tooth loss and three sites of head and neck cancer (oral cavity, pharynx, and larynx). Two studies did not adjust for confounder factors, whereas others controlled a group of conventional risk factors for head and neck cancer, including age, gender, smoking, and drinking.

### Tooth loss and risk of head and neck cancer

**Figure 1:**
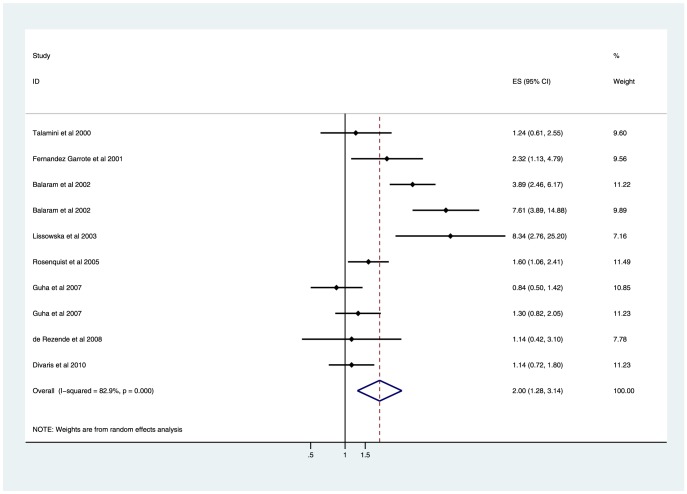
Forest plot showing the association of tooth loss with the risk of head and neck cancer. (Balaram et al 2002 had separate adjusted OR in male and female population.).

A total of 10,722 participations were included in the nine studies exploring the association between tooth loss and risk of head and neck cancer (5,204 assigned to case group and 5,518 assigned to control group). Overall, tooth loss experienced a significant elevated risk for developing head and neck cancer (>5 *vs.* ≤5: OR 2.00, 95%CI 1.28–3.14; *P* = 0.002). Substantial heterogeneity was observed (*I* ^2^ = 82.9%; *P* = 0.000) (**Figure 1**). Subsequently, sensitivity analyses were conducted to explore the potential source of heterogeneity and to examine the effect of various exclusion criteria on the combined risk estimates. Similar results were observed in these analyses, with substantial evidence of heterogeneity (data were shown in **Table 2**).

**Table 2:** Summary of results.

	Studies, N	Cases, N	Control, N	OR, 95%CI	*P*-value	*P* of heterogeneity	I^2^ (%)
>5 *vs.* ≤5							
Total	9	5,204	5,518	2.00 (1.28–3.14)	0.002	0.000	82.9
Assessment of tooth loss							
*Inspected by dentist or interviewer*	7	3,783	4,009	2.31 (1.35–3.97)	0.002	0.000	84.8
*Self-reported*	2	1,421	1,509	1.17 (0.79–1.72)	0.430	0.846	0.0
Adjustment for covariates							
*Yes*	7	5,022	5,148	2.21 (1.26–3..87)	0.006	0.000	86.3
*NA*	2	182	370	1.52 (1.04–2.23)	0.030	0.539	0.0
Sample size							
*Large*	8	5,154	5,468	2.11 (1.31–3.38)	0.002	0.000	84.5
*Small*	1	50	50	1.14 (0.42–3.10)	NA	NA	NA
6–15 *vs.* ≤5							
Total	8	4,613	4,936	1.18 (1.02–1.38)	0.032	0.297	16.8
Assessment of tooth loss							
*Inspected by dentist or interviewer*	6	3,192	3,427	1.24 (1.03–1.50)	0.023	0.179	34.3
*Self-reported*	2	1,421	1,509	1.07 (0.82–1.40)	0.603	0.950	0.0
Adjustment for covariates							
*Yes*	6	4,431	4,566	1.21 (1.02–1.42)	0.024	0.162	36.7
*NA*	2	182	370	1.01 (0.63–1.62)	0.969	0.859	0.0
Sample size							
*Large*	7	4,563	4,886	1.19 (1.01–1.38)	0.032	0.210	28.6
*Small*	1	50	50	1.11 (0.35–3.51)	NA	NA	NA
>15 *vs.* ≤5							
Total	8	4,613	4,936	1.54 (1.08–2.21)	0.018	0.001	73.0
Assessment of tooth loss							
*Inspected by dentist or interviewer*	6	3,192	3,427	1.77 (1.02–3.06)	0.042	0.000	80.1
*Self-reported*	2	1,421	1,509	1.22 (0.96–1.56)	0.100	0.739	0.0
Adjustment for covariates							
*Yes*	6	4,431	4,566	1.39 (0.96–1.56)	0.084	0.004	71.5
*NA*	2	182	370	2.34 (1.47–3.73)	0.000	0.148	52.3
Sample size							
*Large*	7	4,563	4,886	1.59 (1.08–2.33)	0.018	0.000	76.8
*Small*	1	50	50	1.16 (0.40–3.32)	NA	NA	NA

OR, odds ratio; CI, confidence interval; NA, not available; *Large*, cases ≥100; *Small*, cases <100.

### The severity of tooth loss and risk of head and neck cancer

**Figure 2:**
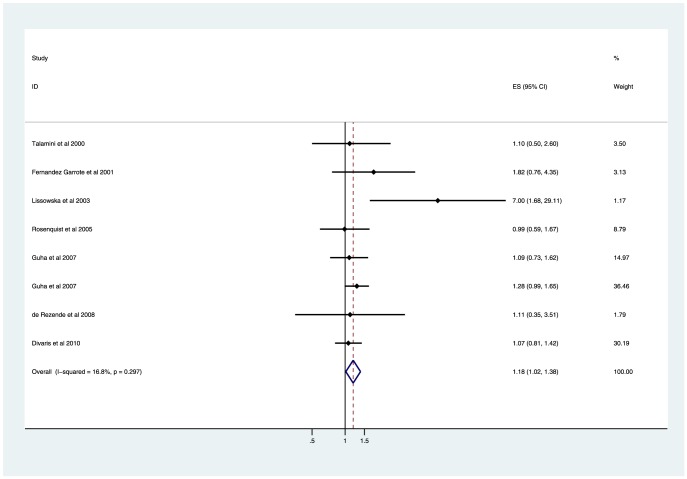
Forest plot showing the association of moderate tooth loss with the risk of head and neck cancer.

**Figure 3:**
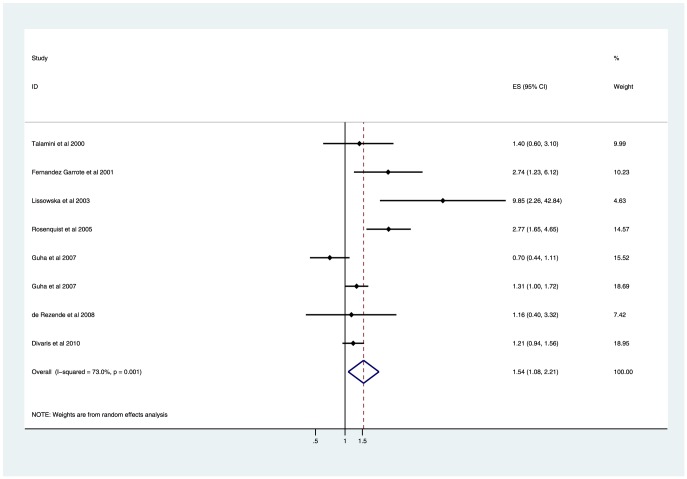
Forest plot showing the association of severe tooth loss with the risk of head and neck cancer.

Furthermore, we conducted meta-analyses based on the grade to explore the effect of the severity of tooth loss on head and neck cancer, and the results were relatively consistent. A significantly increased risk of head and neck cancer was found in both the moderate tooth loss group (6–15 *vs.* ≤5: OR 1.18, 95%CI 1.02–1.38; *P* = 0.032; **Figure 2**) and severe tooth loss group (>15 *vs.* ≤5: OR 1.54, 95%CI 1.08–2.21; *P* = 0.018; **Figure 3**). Substantial evidence of heterogeneity was only observed in the severe tooth loss group (*I* ^2^ = 73.0%; heterogeneity *P* = 0.001), rather than in the moderate tooth loss group (*I* ^2^ = 16.8%; heterogeneity *P* = 0.297). To test the robustness of our findings, sensitivity analyses were performed. Sensitivity analyses yielded similar results, with substantial evidence of heterogeneity (data were presented in **Table 2**).

### Tooth loss and anatomical sites of head and neck cancer

When studies were divided by anatomical sites of head and neck cancer, there was significant increase in larynx cancer (6–15 *vs.* ≤5: OR 1.45, 95%CI 1.14–1.85; *P* = 0.009; *I* ^2^ = 21.0%; heterogeneity *P* = 0.282). However, no association was observed in tooth loss with oral cavity (6–15 *vs.* ≤5: OR 0.92, 95%CI 0.72–1.18; *P* = 0.531; *I* ^2^ = 0.0%; heterogeneity *P* = 0.282) or pharynx cancer (6–15 *vs.* ≤5: OR 1.25, 95%CI 0.94–1.65; *P* = 0.128; *I* ^2^ = 24.1%; heterogeneity *P* = 0.268).

### Publication bias

**Figure 4:**
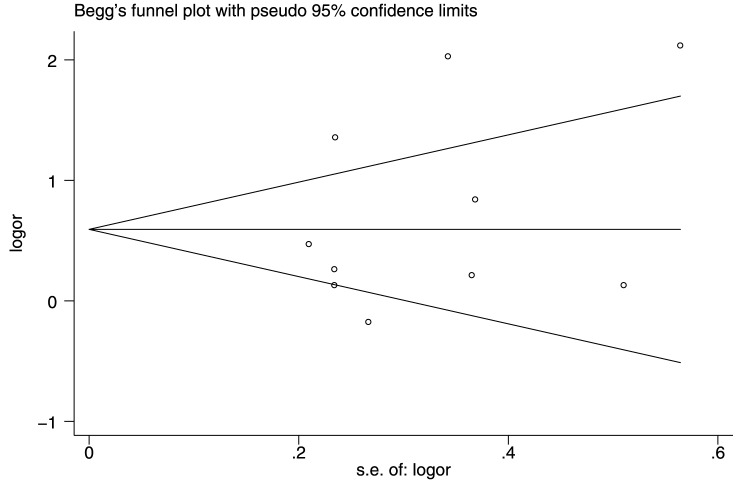
Funnel plots of tooth loss for assessment of publication bias.

Both Begg's (rank correlation test) and Egger's funnel plot asymmetry test (regression method) in the meta-analysis indicated that there was no significant publication bias (>5 *vs.* ≤5: Begg's test, *P* = 0.371; Egger's test, *P* = 0.388; **Figure 4**).

## Discussion

The overall estimates of the present meta-analysis provided evidence that tooth loss was significantly associated with increased risk of head and neck cancer. In addition, the moderate tooth loss and the severe tooth loss experienced a significantly increased risk of head and neck cancer by 18% and 54%, respectively. Furthermore, the moderate tooth loss was associated with a 45% increase in the risk of larynx cancer. The combined estimates were robust across sensitivity analyses and had no observed publication bias.

Several plausible mechanisms may explain why a significant increased association of tooth loss with head and neck cancer was observed in the present analysis. The link between inflammation and cancer has long been recognized [33], [34], [35]. Periodontitis, a chronic inflammatory disease, contribute to constant low-grade systemic inflammation with elevated levels of circulating inflammatory markers [36]. Identified inflammatory markers, including pro-inflammatory plasma cytokines, peripheral white blood cells, prostanoids, proteases including matrix metalloproteinases, and acute-phase proteins [36], [37], [38], produced in the immune response to periodontal disease. Chronic inflammation induced by periodontal pathogens may also result in the breakdown of normal cell growth control and potential carcinogenesis [33]. Alternatively, immune system in an individual with chronic periodontal disease may be deficient at clearing infection, and subsequently deficient at surveillance for tumor growth. Therefore, periodontitis are considered as a marker of a type of immune function that has potential influences on tumor growth and progression. Another plausible mechanism by which may explain the reported observations are increased production of carcinogenic nitrosamines. The formation of endogenous carcinogenic nitrosamines in the oral cavity is elevated by poor oral hygiene, periodontal disease, tobacco smoking, and certain dietary factors [39], [40]. Oral microorganisms also result in greater nitrosamine production [41]. Therefore, drawing a link between tooth loss and head and neck cancer seems plausible. Other mechanisms by which consumptions of alcohol might be risky of head and neck cancer are the acetaldehyde production from alcohol by oral microbiota.

A cohort study on overall cancer risk and tooth loss by Tu et al. suggested that as tooth loss increases, periodontal disease may decrease (as edentulous patients no longer have active periodontal disease) [42]. It was in agreement with another observational study where they found that if the missing teeth were more than 15 the association with esophageal cancer disappeared [25]. That might be a limiting factor in establishing a solid link between tooth loss and head and neck cancer risk. Interestingly, overall risk estimates of the moderate and severe tooth loss did not change such an association of tooth loss with increased head and neck cancer risk (although the strength of the association was slightly attenuated), suggesting that tooth loss is probably an independent risk factor of head and neck cancer. Moreover, sensitivity analyses based other various exclusion criteria did not materially alter the overall estimates, which added robustness to our main finding.

An interesting clue on the topic may be useful for future research. Three case-control studies included in our meta-analysis were conducted to assess the association of tooth loss with the anatomical sites of head and neck cancer. Only two studies consistently suggest that tooth loss significantly increase the risk of larynx cancer rather than oral cavity and pharynx cancer. Thus, a new question arise, does tooth loss really increase the risk of different anatomical sites of head and neck cancer? However, overall risk estimates show that only the moderate tooth loss was associated with a 45% increase in the risk of larynx cancer. The results should be interpreted with caution. There might be several factors ascribing to these contradicting findings. Small numbers of study subjects and included studies were presented in this meta-analysis, which might lower the statistical power of the study by limiting the ability to estimate more precise association. Despite the imprecise estimate, we believe that these associations deserve attention and warrant additional study.

A major strength of the present study is that there was no publication bias, suggesting that such an association is not an artifact of unpublished negative studies. Moreover, the association of tooth loss with risk of head and neck cancer persists and remains statistically significant in sensitivity analyses based on various exclusion criteria, which indicate that our main findings are robust and the association of tooth loss with head and neck cancer is independent of conventional risk factors of head and neck cancer. In addition, with the accumulating evidence and large sample size of included studies, we have great statistical power to provide precise and reliable risk estimates. Despite these advantages, several limitations might be acknowledged in this meta-analysis. First, substantial heterogeneity was observed among studies of tooth loss and head and neck cancer risk, which was not surprising given the differences in characteristics of populations, ascertainment of tooth loss, and adjustment for confounding factors. Despite sensitivity analyses were performed, we were unable to detect the major source of heterogeneity. Noteworthy, the pooled results still turned out to be significant. In addition, residual and unmeasured confounding, such as diabetes, diet changes, and socioeconomic factors, is of concern, which may confound the interpretation of the tooth loss-cancer association and potentially produce biases. Finally, although little evidence of publication bias was observed, the statistical power for these tests was limited due to a relatively small number of included studies.

This meta-analysis suggests that tooth loss significantly increases the risk of head and neck cancer, and the increase is probably independent of conventional head and neck cancer risk factors. Future well-design study is warranted to confirm present findings using more specific measures of tooth loss status, and to examine this postulated association among never smokers. If epidemiologic study confirms the role of tooth loss in cancer etiology, additional studies will be needed to further elucidate the potential biological mechanisms involved.

## Supporting Information

Diagram S1 **The flow diagram of this meta-analysis.**
(DOC)

## References

[1] Ries LAG, Melbert D, Krapcho M, editors (2008) SEER Cancer Statistics Review, 1975–2005. Bethesda, MD: National Cancer Institute. Available: http://seer.cancer.gov/csr/1975_2005.

[2] Jemal A, Siegel R, Ward E, Hao Y, Xu J, et al (2008) Cancer statistics, 2008. CA Cancer J Clin 58: 71–96. 18287387 10.3322/CA.2007.0010

[3] Marur S, Forastiere AA (2008) Head and neck cancer: changing epidemiology, diagnosis, and treatment. Mayo Clin Proc 83: 489–501. 18380996 10.4065/83.4.489

[4] Parkin DM, Bray F, Ferlay J, Pisani P (2005) Global cancer statistics, 2002. CA Cancer J Clin 55: 74–108. 15761078 10.3322/canjclin.55.2.74

[5] Blot WJ, McLaughlin JK, Winn DM, Austin DF, Greenberg RS, et al (1988) Smoking and drinking in relation to oral and pharyngeal cancer. Cancer Res 48: 3282–3287. 3365707

[6] Zheng TZ, Boyle P, Hu HF, Duan J, Jian PJ, et al (1990) Dentition, oral hygiene, and risk of oral cancer: a case-control study in Beijing, People's Republic of China. Cancer Causes Control 1: 235–241. 2102296 10.1007/BF00117475

[7] Gillison ML, Lowy DR (2004) A causal role for human papillomavirus in head and neck cancer. Lancet 363: 1488–1489. 15135592 10.1016/S0140-6736(04)16194-1

[8] Balaram P, Sridhar H, Rajkumar T, Vaccarella S, Herrero R, et al (2002) Oral cancer in southern India: the influence of smoking, drinking, paan-chewing and oral hygiene. Int J Cancer 98: 440–445. 11920597 10.1002/ijc.10200

[9] Schildt EB, Eriksson M, Hardell L, Magnuson A (1998) Oral infections and dental factors in relation to oral cancer: a Swedish case–control study. Eur J Cancer Prev 7: 201–206. 9696928 10.1097/00008469-199806000-00004

[10] Albandar JM, Brunelle JA, Kingman A (1999) Destructive periodontal disease in adults 30 years of age and older in the United States, 1988–1994. J Periodontol 70: 13–29. 10052767 10.1902/jop.1999.70.1.13

[11] Pihlstrom BL, Michalowicz BS, Johnson NW (2005) Periodontal diseases. Lancet 366: 1809–1820. 16298220 10.1016/S0140-6736(05)67728-8

[12] Papapanou PN (1996) Periodontal diseases: epidemiology. Ann Periodontol 1: 1–36. 10.1902/annals.1996.1.1.1 9118256

[13] Marshall JR, Graham S, Haughey BP, Shedd D, O'Shea R, et al (1992) Smoking, alcohol, dentition and diet in the epidemiology of oral cancer. Eur J Cancer B Oral Oncol 28B: 9–15. 1422474 10.1016/0964-1955(92)90005-l

[14] Day GL, Blot WJ, Austin DF, Bernstein L, Greenberg RS, et al (1993) Racial differences in risk of oral and pharyngeal cancer: alcohol, tobacco, and other determinants. J Natl Cancer Inst 85: 465–473. 8445674 10.1093/jnci/85.6.465

[15] Bundgaard T, Wildt J, Frydenberg M, Elbrond O, Nielsen JE (1995) Case-control study of squamous cell cancer of the oral cavity in Denmark. Cancer Causes Control 6: 57–67. 7718736 10.1007/BF00051681

[16] Tezal M, Grossi SG, Genco RJ (2005) Is periodontitis associated with oral neoplasms? J Periodontol 76: 406–410. 15857075 10.1902/jop.2005.76.3.406

[17] Tezal M, Sullivan MA, Reid ME, Marshall JR, Hyland A, et al (2007) Chronic periodontitis and the risk of tongue cancer. Arch Otolaryngol Head Neck Surg 133: 450–454. 17515503 10.1001/archotol.133.5.450

[18] Hiraki A, Matsuo K, Suzuki T, Kawase T, Tajima K (2008) Teeth loss and risk of cancer at 14 common sites in Japanese. Cancer Epidemiol Biomarkers Prev 17: 1222–1227. 18483345 10.1158/1055-9965.EPI-07-2761

[19] Michaud DS, Liu Y, Meyer M, Giovannucci E, Joshipura K (2008) Periodontal disease, tooth loss, and cancer risk in male health professionals: a prospective cohort study. Lancet Oncol 9: 550–558. 18462995 10.1016/S1470-2045(08)70106-2 PMC2601530

[20] Tezal M, Sullivan MA, Hyland A, Marshall JR, Stoler D, et al (2009) Chronic periodontitis and the incidence of head and neck squamous cell carcinoma. Cancer Epidemiol Biomarkers Prev 18: 2406–2412. 19745222 10.1158/1055-9965.EPI-09-0334

[21] Garrote LF, Herrero R, Reyes RM, Vaccarella S, Anta JL, et al (2001) Risk factors for cancer of the oral cavity and oro-pharynx in Cuba. Br J Cancer 85: 46–54. 11437401 10.1054/bjoc.2000.1825 PMC2363910

[22] Talamini R, Vaccarella S, Barbone F, Tavani A, La Vecchia C, et al (2000) Oral hygiene, dentition, sexual habits and risk of oral cancer. Br J Cancer 83: 1238–1242. 11027440 10.1054/bjoc.2000.1398 PMC2363583

[23] Lissowska J, Pilarska A, Pilarski P, Samolczyk-Wanyura D, Piekarczyk J, et al (2003) Smoking, alcohol, diet, dentition and sexual practices in the epidemiology of oral cancer in Poland. Eur J Cancer Prev 12: 25–33. 12548107 10.1097/00008469-200302000-00005

[24] Rosenquist K, Wennerberg J, Schildt EB, Bladstrom A, Goran Hansson B, et al (2005) Oral status, oral infections and some lifestyle factors as risk factors for oral and oropharyngeal squamous cell carcinoma. A population-based case-control study in southern Sweden. Acta Otolaryngol 125: 1327–1336. 16303683 10.1080/00016480510012273

[25] Guha N, Boffetta P, Wunsch Filho V, Eluf Neto J, Shangina O, et al (2007) Oral health and risk of squamous cell carcinoma of the head and neck and esophagus: results of two multicentric case-control studies. Am J Epidemiol 166: 1159–1173. 17761691 10.1093/aje/kwm193

[26] Rezende CP, Ramos MB, Daguila CH, Dedivitis RA, Rapoport A (2008) Oral health changes in with oral and oropharyngeal cancer. Braz J Otorhinolaryngol 74: 596–600. 18852988 10.1016/S1808-8694(15)30609-1 PMC9442110

[27] Divaris K, Olshan AF, Smith J, Bell ME, Weissler MC, et al (2010) Oral health and risk for head and neck squamous cell carcinoma: the Carolina Head and Neck Cancer Study. Cancer Causes Control 21: 567–575. 20049634 10.1007/s10552-009-9486-9 PMC2925153

[28] Higgins JP, Thompson SG, Deeks JJ, Altman DG (2003) Measuring inconsistency in meta-analyses. BMJ 327: 557–560. 12958120 10.1136/bmj.327.7414.557 PMC192859

[29] DerSimonian R, Laird N (1986) Meta-analysis in clinical trials. Control Clin Trials 7: 177–188. 3802833 10.1016/0197-2456(86)90046-2

[30] Alexander J, Sutton KRA, Jones DR, Sheldon TA, Song F (2000) Methods for meta-analysis in medical research. Wiley Chichester

[31] Begg CB, Mazumdar M (1994) Operating characteristics of a rank correlation test for publication bias. Biometrics 50: 1088–1101. 7786990

[32] Egger M, Davey Smith G, Schneider M, Minder C (1997) Bias in meta-analysis detected by a simple, graphical test. BMJ 315: 629–634. 9310563 10.1136/bmj.315.7109.629 PMC2127453

[33] Coussens LM, Werb Z (2002) Inflammation and cancer. Nature 420: 860–867. 12490959 10.1038/nature01322 PMC2803035

[34] van Kempen LC, de Visser KE, Coussens LM (2006) Inflammation, proteases and cancer. Eur J Cancer 42: 728–734. 16524717 10.1016/j.ejca.2006.01.004

[35] Karin M, Lawrence T, Nizet V (2006) Innate immunity gone awry: linking microbial infections to chronic inflammation and cancer. Cell 124: 823–835. 16497591 10.1016/j.cell.2006.02.016

[36] Moutsopoulos NM, Madianos PN (2006) Low-grade inflammation in chronic infectious diseases: paradigm of periodontal infections. Ann N Y Acad Sci 1088: 251–264. 17192571 10.1196/annals.1366.032

[37] Oringer RJ (2002) Modulation of the host response in periodontal therapy. J Periodontol 73: 460–470. 10.1902/jop.2002.73.4.460 11990448

[38] Loos BG (2005) Systemic markers of inflammation in periodontitis. J Periodontol 76: 2106–2115. 10.1902/jop.2005.76.11-S.2106 16277583

[39] Nair J, Ohshima H, Nair UJ, Bartsch H (1996) Endogenous formation of nitrosamines and oxidative DNA-damaging agents in tobacco users. Crit Rev Toxicol 26: 149–161. 8688158 10.3109/10408449609017928

[40] Risch HA (2003) Etiology of pancreatic cancer, with a hypothesis concerning the role of N-nitroso compounds and excess gastric acidity. J Natl Cancer Inst 95: 948–960. 12837831 10.1093/jnci/95.13.948

[41] Abnet CC, Qiao YL, Dawsey SM, Dong ZW, Taylor PR, et al (2005) Tooth loss is associated with increased risk of total death and death from upper gastrointestinal cancer, heart disease, and stroke in a Chinese population-based cohort. Int J Epidemiol 34: 467–474. 15659476 10.1093/ije/dyh375

[42] Tu YK, Galobardes B, Smith GD, McCarron P, Jeffreys M, et al (2007) Associations between tooth loss and mortality patterns in the Glasgow Alumni Cohort. Heart 93: 1098–1103. 17164486 10.1136/hrt.2006.097410 PMC1955024

