# Supplementary material for: Tooth Loss and Risk of Head and Neck Cancer: A Meta-Analysis
Source: PLoS One. 2013 Aug 19;8(8):e71122. doi: 10.1371/journal.pone.0071122 (PMC3747175; doi:10.1371/journal.pone.0071122)
Supplement: Diagram S1 — The flow diagram of this meta-analysis. (DOC) [file pone.0071122.s001.doc]

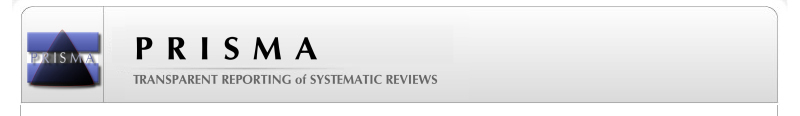
**PRISMA 2009 Flow Diagram**

**Screening**

**Included**

**Eligibility**

**Identification**

Records identified through English database

(PubMed n =2993, Embase n=576)

Additional records identified through hand-search
(n = 4)

Records after duplicates removed
(n = 2,997)

Records screened
(n = 39)

Records excluded
(n = 22)

Full-text articles assessed for eligibility
(n = 17)

Full-text articles excluded, with reasons
(n = 9):

3 Data unavailable

6 Not with ≤5 lost teeth as the referent category

Studies included in qualitative synthesis
(n = 8)

Studies included in quantitative synthesis (meta-analysis)
(n = 9 )
